# Collectanea

**Published:** 1826-09

**Authors:** 


### COLLECTANEA.

Floriferus, ut apes, in saltibus omnia libant,

Omnia nos, itidem, depascimur aurea dicta.

### PHYSIOLOGY.

### *Uterus wanting.*

M. RENALDIN lately examined the sexual organs of a female, in whom the uterus was wanting. The woman, aged fifty-two, died from cancerous affection of the stomach; she was very small, and did not exceed in height three and a half feet; her understanding was very little developed; she had never menstruated, and her breasts had never enlarged. The parts of generation were externally well formed; the hymen still partly existed. On introducing the finger deeply into the vagina, there was felt, instead of the neck of the uterus, a small tubercle, scarcely perceptible. Between the bladder and rectum there was, instead of the uterus, a sort of resisting cord, of the thickness of a writing-quill, communicating at one end with the vagina, at the other with the fallopian tubes: these, much widened at the point where they entered the canal, formed there a sort of little sac. There were scarcely any remains of ovaries. On opening the vagina and the little canal at the end of it: the first was found sufficiently developed, and the other, which was an inch long, was evidently, by its consistence and organisation, the neck of the uterus imperfectly formed, the body and fundus of that organ being entirely wanting. (*Revue Medicale.*)

### *Acephalous Monster.*

M. Andral, fils, read, at the Academie Royale de Medecine, the report of a case of an acephalous, sent by M. Allauneau, a physician at Thouars. The subject of this case is a fœtus of eight months, still born, and without any vestige of brain or spinal marrow, in whom the cavities of the brain and spinal marrow contained only cellular tissue. The bony parts forming the vault of the cranium were very imperfectly developed : for example, of the frontal and occipital bones, there was only an orbitar and basilar portion. This confirms the statement of M. G. St. Hilaire, that, when the nervous mass is wanting, the bones destined to cover it are wanting also. On the contrary, however, all the nerves were present; but the author has forgot to mention, with sufficient precision, their termination in the cranium and spinal canal. This, then, is another fact to add to those which prove already the possibility of the non-existence of the brain and spinal cord in a fœtus nearly of the full time: but it does not throw any light on the question agitated—whether, in these acephalous monsters, the nervous centres have ever existed? or if, having existed at first, they have been destroyed by some accidental cause? Morgagni was of this last opinion; but the chief anatomists of the present day profess the former: first, because the growth of the embryo proves that the nerves develope themselves, not from nervous centres to the organs on which they are distributed, but from those organs to the nervous centres; and, secondly, because comparative anatomy has shown that there are certain fishes whose spinal nerves have no connexion with the spinal marrow, and are separated from it by a liquid. (*Ibid.*)

### *Monster, in whom the Extremities were entirely wanting.*

Dr. Hastings gives the following description of this monster:

*Head.*—There is a great quantity of black hair on the head, and the bones are more perfect than they are usually found at birth. The nose is very prominent, and there is a red mark upon it, which extends to the upper lip. The fontanelles are rather small.—*Trunk:* The spine appears perfect, and so are the ribs. All the bones seem larger than they usually are at birth. There is a clavicle and scapula on each side, with muscles attached, which move the scapulæ freely. There is an acromion to each scapula. The glenoid cavities are absent. The clavicle is united as usual to the sternum and acromion. The sternum is natural.—*Pelvis:* The sacrum and os coccygis are natural. Ossa innominata appear perfect in every respect, excepting the acetabulum, which is wanting. On the right side there is not the least attempt at the production of a lower extremity; but on the left side, at the part of the ossa innominata into which the head of the thigh-bone is generally received, there is a little process in the shape of a finger, consisting of two small bones united by ligaments, and covered with skin and cellular membrane. This projection apparently has muscles attached to it, as it is constantly moved by the cries or struggles of the child. The projection is thick at the base, and runs to a point, and there is a nail affixed to it. The length of the projection is one inch and eight-tenths. The child cries loudly; it is fed, as the mother cannot suckle it.

It was born on the 9th of April, 1824, and died in October of the same year, of inflammation of the lungs. (*Med.-Chirurgical Transactions of Edinburgh*, vol. ii.)

---

### PATHOLOGY.

### *Observations on the Blood.*

M. Segalas lately read, at the Academie de Medecine, a Memoir on the Blood, which he considers to be susceptible of morbid alteration during life, thus becoming a frequent cause of disease. The following experiments are adduced as proofs of this opinion:

1st. That concentrated alcohol acts chemically on the blood during life.*

2d. Diluted alcohol, injected into the veins or bronchia, produces immediate intoxication; the same effect is induced when it is applied to other parts, but more slowly.

3d. The effects of alcohol, introduced into any other part than the veins, are, in their intensity and quickness, in direct relation with the absorbing powers of that part, and are independent of the nerves distributed there, especially of those of the stomach.

4th. That the effects are hastened, increased, or retarded and diminished, by the circumstances which favour or impede the entrance of alcohol into the blood.

5th. That inebriation vanishes as alcohol leaves the blood, and this effect depends on the circumstances being more or less favourable to exhalation.

6th. That the effects of alcohol are in relation, not with the quantity *introduced* into the organs, but with the quantity *in* the blood.

7th. That profound intoxication, and death by drunkenness, are attended with an evident alteration of the blood, and some changes of the solids less evident.

M. Segalas infers from the above theory, that oil and ammonia are useful in drunkenness; perhaps, they exert an action on the blood opposed to that of alcohol. (*Archives Generales.*)

* The same happens after death; but this action is equally exerted by other chemical agents in producing coagulation of its albumen.

### PRACTICAL MEDICINE.

### *Clinical Report of Medical Cases treated at the Hôtel Dieu.*

In the Clinical Report of the practice at the Hôtel Dieu, speaking of the Sulphate of Quina, M. Martinet says, its action is very variable in different persons. He particularly mentions the case of a man who had a quartan ague for five weeks, and with whom four grains of the salt sufficed to stop the fever completely; the ague was caught in Paris, although the man had for some months before inhabited a marshy country without being seized with it. Although many physicians administer the sulphate of quina in very small doses, (as two, four, or six grains,) and that in the fear of augmenting or provoking any internal inflammation, he remarks, that, with some people, the only means of getting the full benefit of the medicine is to give it in doses of *twelve, sixteen, twenty, or twenty-four grains*; and that in this way only we can immediately cut short the fever.

Among the diseases of the chest, there is a case of loss of voice, apparently dependent on irregular menstruation. A young woman, nineteen years old, had not menstruated for two months, when, towards the end of December, she was seized with pain corresponding to the middle of the sternum, with slight fever. Next day she came to the hospital with these complaints, and nearly entire loss of voice; the breathing was free. She was bled; the fever left her, but the loss of voice continued. At times this patient complained of colicy pains, headaches, and palpitations, which were considered to be dependent on the amenorrhœa, and were treated, without advantage, by applications of leeches to the vulva, and a second bleeding. A blister on the neck did no good to the state of her voice, which, on the contrary, became worse, and at length was entirely lost. The patient had no cough, no spitting; did not lose flesh, and was able to walk about the wards; her breathing was free, when she kept herself quiet, but exertion brought on palpitation and dyspnœa. This state continued two months, when, on March 3d, 1826, the menses appeared naturally. The next day at the visit, to the astonishment of all, she had recovered the use of her voice, although it was yet very feeble. Nature has evidently all the merit of this recovery, yet had any particular remedy, as magnetism or electricity, been used at the time, the whole credit of the cure would have been given to it. This case shows that we should never despair of the restoration of a sense or faculty, when there is no destruction of the organ to account for its loss.

Catarrhal fevers, with gastric symptoms, were frequent, and required the use of purges, diluents, &c. Six cases, where there was white yellow coating of the tongue, bitter taste in the mouth, anorexia, and headaches, were treated in this way by M. Recamier. In other six there was a slight phlogosis of the digestive canal, characterised by abdominal pain and slight diarrhœa:

the same mode of treatment was equally successful. Five cases, in which there were symptoms of what the writer calls *catarrhal phlogosis*, without gastric symptoms, the tongue being sometimes red, at others white and papillous, were treated with leeches on the epigastric and iliac regions. This mode of treatment was neither quicker nor more successful than the other.

With regard to the redness of the tongue, and the form it takes, M. Recamier cautions us that it is very necessary, in order to judge exactly of its colour, to observe the manner in which patients put it out. *He thinks the tongue blushes like the face, in consequence of a moral impression*, and that the presence of the physician sometimes produces this effect; whence the error into which a person may be led, who hastily states the tongue to be red.

In one patient, leeches to the anus were useful: a diarrhœa of ten days' duration was stopped by them. In three cases M. Recamier began with small doses of tartar emetic, and then had recourse to general bleedings, to calm the intensity of the fever: they got better like the others, and without any thing particular occurring. It was not so in a young man, aged twenty-one, a mason, who for eight days suffered pain in the region of the cœcum, with high fever, headache, white tongue, constipation, and symptoms of pulmonary catarrh: he was twice bled without benefit; there was even commencing stupor and great prostration of strength; a brownish spot showed itself at the inner part of the arm; the surrounding skin became red, tense, and swollen. Several applications of leeches did not arrest the progress of the inflammation: the brown spot increased; the neighbouring skin became greyish and gangrened, as well as the superficial muscles. Deep cauterisation arrested its progress, but it had already so extended as to occupy all the inner surface of the left arm, and almost the whole of the forearm. When the eschar dropped off, the wound was ten inches long and five broad. In three months and a half perfect cicatrisation took place, and the young man went away cured.—M. Recamier remarked in his lecture, that the erysipelatous phlegmon on the arm had followed the gangrenous spot spoken of, and that consequently the eschar had not been the effect of inflammation. He was confirmed in this opinion from the want of connexion between the puncture made in bleeding and the gangrenous spot, which was more than an inch from it, and from the simultaneous occurrence of a similar appearance in two patients then in the wards; one of whom had partial gangrene of the hand, the other an enormous eschar of the leg and foot, where no previous inflammation could be discovered. M. R. gave it as his opinion, that it was a case of gangrene from a specific cause, as is observed in malignant pustule, save that in the cases just mentioned the morbific principle, instead of having an external origin, originated in the patient himself. Such are the Professor's opinions on the origin of these spontaneous gangrenes, which occasionally come on during the course of severe catarrhal and typhoid fevers, and of which they are often the fatal crises.

A woman, two days after her confinement, had pain in the left groin, extending to the lower belly. Some applications of leeches removed the swelling of the integuments, and the pain ceased almost entirely; but this improvement did not last long: the pains reappeared, and with them fever, headache, and towards the end diarrhœa. In spite of leeches to the tumor, baths, and the opening of a small abscess, which showed itself externally, she daily got worse, and died shortly after. On opening the body, an abscess, of the size of a hen's egg, was found situated in the pelvis, in the neighbourhood of the third ligament of the left side, and extending to the bladder, womb, and rectum. Besides this, the colon was ulcerated.

Five patients, with acute rheumatic affections of the joints, were treated with the tartar emetic, in doses of six to eight grains in four ounces of liquid, to which was added half an ounce of syrup. The antimony given thus, seldom caused vomiting, but produced abundant stools: the tongue preserved its moisture, and took on no unnatural redness. In general, this plan was followed by relief; the swelling and pain abated, and the duration of the disease was curtailed. He does not say so much for opium, which was given in mass, to some patients, in doses of from two to four grains: it had no effect on the rheumatism; one only experienced from it a slight sleepiness, which soon went off. (*Revue Medicale.*)

### *A new Mode of Treatment in Herpes Zoster.*

M. Serres has lately discovered that the ectrotic plan, used with success in small-pox, has great efficacy in relieving the pain sometimes so severe in shingles. He was led to this discovery by the analogy existing between this disease and small-pox. He relates several cases where cauterising with the nitras argenti had a good effect, and he strongly recommends the use of this means in severe cases. (*Ibid.*)

---

### STATISTICAL MEDICINE.

### *Variolous Epidemic in Sweden.*

The last Number of Magendie's Journal of Physiology contains a notice of the variolous epidemic which prevailed very generally throughout the provinces of Sweden in 1824, and the early part of 1825. The details are abstracted (by Dr. De Fermon) from the official Reports collected and published by the College de Santé of Stockholm; and we regret that our limits will not permit us to give more than a brief sketch of the principal features of this very interesting Memoir. It is introduced into the French Journal by way of appendix to Dr. Gregory's paper on Small-Pox, which was published in the February Number of this Journal; and the French editors appear to have been struck by the coincidence in the results of the different observations.

We are informed that, since the year 1801, small-pox was never so general in Sweden as to attract attention, until May 1823, when it was brought to Gottenburg by a negro from Amsterdam. From thence it spread to most of the provinces, and ultimately to Stockholm. By the middle of 1825, the epidemic had totally ceased. Among the several Reports addressed to the Stockholm College by the country practitioners, those of M. Hedlund of Hernœsand, M. Nordblad in the district of Helsingland, and M. Robsahm in that of Western Gottiland, are specially mentioned. They all appear to have been accurate and unprejudiced observers, and the following may be taken as the general results of their Reports.

The total numbers that died of small-pox in Sweden during the *two* years preceding the epidemic, were only 23. In 1824 (the year of the epidemic), the deaths amounted to 560; of whom, 229 were children under two years of age, 162 between the ages of two and fifteen, and 169 adults. Of this number, 34 were *ascertained* to have been vaccinated, and 69 are noted as doubtful. Most of them were of the lower orders, and, with the exception of four, above fifteen years of age; rendering it probable, as the Memoir observes, that they were vaccinated at a distant period, when the process was less carefully conducted than at present. In the more detailed account which is given of the practice of Messrs. Hedlund and Nordblad, it is obvious that great difficulties were experienced in determining the *efficiency* of the original vaccination. No particular notices, however, are given of the appearance of the scars, either in the mild or fatal cases.

M. Hedlund observes, that the epidemic assumed three different forms,—viz. genuine small-pox, varicella (*watt-koppor*), and fever without eruption. In proof of the connexion of the *latter* with the contagion of small-pox, he asserts that it resembled exactly the initiatory fever of true variola, and that the affection began and ceased with the other forms of the epidemy. He estimates the numbers attacked by this varioloid *fever* at one-third of those who suffered under the two other forms which the epidemic assumed.

It is gratifying to perceive that the ultimate result of the epidemic was decidedly favourable to the cause of vaccination. Wherever the correctness of the process was clearly and indisputably ascertained, the succeeding disease is said to have *hardly deserved the name of small-pox*. No attempt, however, was made to conceal or to explain away the unsuccessful cases. The facts are simply stated, and the public permitted to draw its own judgment from the general course and tenor of the disease. A table is given of the total mortality occasioned by small-pox in Sweden during the last fifty years, by which it can be made clearly to appear that, in that country alone, vaccination has been the means of saving seventy-two thousand lives!

We think that this brief notice of the Swedish epidemic may not be without its use in this country. It has been the frequent custom of writers to throw in our teeth the practice of foreign countries, in many of which it is said small-pox has long ceased to exist. In the very last Report of the Vaccine Board, for instance, we read "that, in many parts of the continent, where the method inculcated by this Board has been adopted, the small-pox may be said to be almost, if not altogether, extirpated." The Memoir, of which we have now presented an abstract, may be received as a proof that our neighbours are not a whit better off than ourselves; and that, if the small-pox be not actually among them, the *seeds* of it at least are there, which the accidental landing of a negro may quicken into a formidable epidemic. We think, too, that some of our authorities may take another lesson from our Swedish neighbours, and not consider the good cause in danger because a few persons have been declared to die of small-pox after vaccination. Here we see the Swedish College announcing *thirty-four* deaths after vaccination, without any idea that, by so doing, they are injuring it in public estimation. Such events, in truth, must naturally be expected whenever an epidemic small-pox visits any great country, considering what an infinite number of persons are engaged in vaccinating, and how difficult it is, with every possible care, (at least in our present state of knowledge,) to determine the real extent to which the system of any individual has been saturated by the vaccine process.

### *Vaccination.*

Dr. J. Grabner Maraschim, in an Essay published by him on Small-pox modified by Vaccination, states his opinion that vaccination should be renewed every ten years. He has made experiments on the subject, the result of which was, that, in twenty-eight subjects vaccinated by him *after* ten years from the first vaccination, twenty-one showed unequivocal signs of again having the true vaccine disease. The lymph of these was used to vaccinate those who had never been vaccinated, and with complete success. To complete his experiments, he vaccinated fifteen subjects who had been previously vaccinated *within* ten years, and in all of them the inoculation was without effect.

---

### SURGERY.

### *Operation for Imperforate Anus.*

This case was the son of Mr. Smith, a tinman, residing at No. 43, Whitcomb-street, and had been born forty-eight hours when the operation was performed, on the 17th November, 1822.

The raphe was the only guide we had for the operation, there being neither hollow nor depression to mark the spot where nature had failed in completing her design. Dr. Granville kindly assisted me at the operation, by securing the child, and by keeping its lower extremities in a proper position during the operation, which was done by making an incision, about an inch and a half in length, with the scalpel, through the skin and fat, nearly as deep as the incision was long, but narrowing it two-thirds at its fundus. Not having reached the intestine with the scalpel, and considering that we could not so safely proceed further upwards in the direction of the gut with that instrument as with the trocar, the latter was preferred, and directed gently upwards, backwards, and inclining to the direction of the sigmoid flexure of the colon for about an inch; when, on withdrawing the stilette, we found that the intestine had not yet been reached: the stilette was therefore again passed through the canula, which was still kept in the parts, and pushed upwards half an inch farther, when, from a want of resistance, I suspected we had at length succeeded, and, on withdrawing the stilette a second time, meconium flowed through the canula in considerable quantity: and here it was curious to witness the instinctive straining of the child to relieve itself for the first time, and which would suggest the advantage to be derived from the practice of gently irritating the external skin over the situation of the anus in such cases, with a view of ascertaining the probable distance of the gut from the surface, as before noticed; for, on this occasion, the contorted features of the infant were precisely like those of an adult who was constipated, and straining to relieve himself.

The canula was secured by tapes, and retained in the parts three days. It was then withdrawn, cleaned, and again introduced, the fæces passing through it during that period.

After about a week or ten days, the canula was removed, and an old-made sponge tent introduced in its stead; but, whether from its age, or from there being too much wax in its composition, it did not expand, and consequently did not dilate the parts. Some sponge tents recently made were also tried, and likewise laid aside, from their inefficiency. The common smooth-made bougie, of the largest size, was, after some weeks, substituted, and was found to answer the purpose much better. The tents used were about three inches and a half in length, and, as they were introduced close up to their thickest extremity, we ascertained precisely the distance of the intestine from the surface, by measuring the tent with a scale; the end of the part tinged with bile indicating the termination of the gut, and the verge of the newly-formed anus marking the length of the artificial canal, and which we found to be exactly three inches.

The child's bowels were occasionally constipated for two or three weeks; but this was as frequently obviated by the administration of small doses of the ol. ricini. Two months had now elapsed from the operation, when the mother was directed to introduce the bougie for a few hours only every day, and I then took my leave.

At the end of three months, the mother brought the child to my house, and stated that, although its bowels were regular, and the usual quantity of fæces evacuated, she had that morning observed, for the first time, that its urine was in some degree tinged with fæces; but, on being further questioned, she stated that she had no reason to believe the urine ever passed per anum. The child fed well, grew, was healthy, and some teeth appeared at the usual period; yet still the urine continued to be occasionally tinged with fæces, and, until the morning of the day on which it died, the 29th September, 1823, (being more than ten months after the operation,) I heard of no one circumstance to lead me to suppose that the child had been otherwise than well.

On examining this child post mortem, the artificial anus was found situated in a hollow, so precisely as if it had been originally natural, that the best anatomist would have been deceived by it: and this fact is the more worthy of record, when it is borne in mind that, at the period the operation was performed, there did not appear the smallest depression or fissure on any part along the line of the raphe, both nates preserving a continuous convex surface.

When the abdominal contents were exposed to view, by reflecting the divided parietes, a somewhat extraordinary disposition of the bowels presented itself; for the small intestines were, apparently, all lying on the left side, resting on the sigmoid flexure of the colon; the intestinum rectum was very large, and distended with air, being at its widest part (viz. five inches above the external aperture, or artificial anus,) *six inches and three-fourths in circumference;* and, passing from the centre above the pubes to the right side, it rested upon the cœcum caput coli, and then turned downwards behind the bladder to the artificial anus. The lower part of the rectum adhered to the bladder by its peritoneal reflexion in the usual manner. A small valvular aperture was discovered between the urethra and rectum. (Mr. Copland Hutchison's *Observations in Surgery.*

*Large Calculus extracted from the Bladder of a Child.*

In the neighbourhood of Lucignano, a young female was delivered of a male child, which was very small and extenuated, although the mother had completed the usual term of pregnancy. From time to time the infant continued to scream violently, and these attacks were followed by intervals of ease. The bowels were at first thought to be the seat of disease, and many purgatives prescribed in consequence. At length the mother observed that these screams were connected with the passing of urine, which was performed with so much straining that a prolapsus of the anus was produced by it. When the child reached its third year, the spasms, in passing even a single drop of water, became more violent, occurring every eight or ten minutes. When first seen by the relater of the case, (June 1825,) the child was much reduced in flesh; there was a prolapsus of the rectum, to the extent of two inches; and the part was much swollen and indurated.

The presence of a stone in the bladder being ascertained, the next day the lateral operation was performed. The introduction of the sound was very easily accomplished, and the incision into its groove performed with the usual facility; but the operator was then greatly surprised on feeling with the index finger a considerable cavity, of an irregular and unequal surface ; from whence it appeared that the sound was in reality not in the bladder, but situated between it and the rectum. The discharge of a bloody matter convinced the operator that this was a false opening, in a state of suppuration; and it appeared, upon questioning the mother, that, some weeks prior to the operation, a surgeon had attempted forcibly to introduce a large catheter, not at all adapted to the child's age, had produced a good deal of hemorrhage, and, not meeting with any foreign body, fancied he had penetrated into the bladder. There was some difficulty in finding the right opening into the bladder; but, when that was overcome, the operation was finished in the usual manner, and the child recovered without any accident. The rectum, after the operation, gradually became flaccid, and finally retired into its natural situation.

The stone was of an oval form, a little flattened on the sides; its greatest diameter was one inch seven lines, the smaller not quite an inch; its surface was smooth. (*Annali Universali.*)

*Ununited Fracture of both Bones of the Fore-arm, cured by the Excision of the Ends of the Bone.*

A countryman had both bones of the fore-arm broken by a blow with a stick, in October 1819. The fracture was united at the end of a month, but, the man returning too soon to his work, the fracture gave way again. In January 1820 attempts were again made, by proper bandages, &c. for two months, to procure a consolidation of the fractured parts, but without effect. The surgeon, suspecting that the fracture was oblique, and that the union might have taken place in a few points, while the rest of the bone was covered with a fibro-cartilaginous substance, proceeded to rub the ends of the bone together, in the hope of producing adhesive inflammation; but, after another trial of three months, this was found ineffectual. An operation was therefore determined upon, which was performed on the 13th June, and about three lines of the ends of the ulna removed; which bone was fractured obliquely, as had been suspected. The man refused, however, to have any thing done to the radius, the previous operation having been very tedious and painful.

Great inflammation succeeded to the operation, and matter formed not only at the part, but in other situations of the fore-arm, requiring several counter openings to be made. At the end of a month, however, it was found that both bones were perfectly united; and the man, at the end of some months, was enabled to resume his usual avocations. (*Ibid.*)

*A Case of Abscess of the Antrum Maxillare, attended with extensive Caries of Bone.* By Dr. J. De la Motta.

An abscess in the antrum maxillare burst in the mouth, "over the palatine bones," and the face, particularly under the eye, became considerably swelled: a poultice was applied. Stupor and vertigo came on. An opening was made in the mouth, which gave vent to about an ounce of matter. Some days after, matter pointed below the external angle of the eye; the os malæ was diseased. An orifice was made, enlarged, and the malar bone exfoliated, and continued to do so for a long time. The palatine plates, on the left side, exfoliated. Two teeth were extracted, and matter passed by their sockets.

A probe, introduced through the orifice made at the external angle of the orbit, could be passed into the mouth, through the opening made by the removal of the teeth. A seton was inserted, following the tract above described; and, although this plan of treatment has been reprobated, and its utility impeached, still in this instance it evidently aided in the speedy removal of the carious portion of bone. After it was withdrawn, the discharge continued, attenuated: a strong injection of sulphate of zinc was used, which, being steadily pursued, produced a healthy change. The external and internal orifices soon closed; and, with the exception of a very inconsiderable discharge from the nostril of the affected side, the patient got well, having a depression about the lower lid, much more considerable than is observed on the opposite side. (*Carolina Journal of Medicine.*)

---

### MIDWIFERY.

### *New Method of Extracting the Placenta.*

Dr. Mojon, Emeritus Professor in the University of Genoa, has communicated, through the Giornale Critico di Medicini Analitica, a new method of extracting the placenta from the uterus, in cases of severe hemorrhage after delivery. The method consists in the injection, by a syringe, with some force, into the placenta, through the veins of the umbilical cord, of cold water, slightly acidulated with vinegar; after having first withdrawn as much blood as may be possible from this vein. The sudden impression, and the tendency to separation, which this injected water produces on the tissue of the placenta, – the cold which is instantaneously conveyed by it to the fleecy portion uniting it with the womb, by which the tendency to contraction in the womb is increased,—and the greater weight which the placenta itself acquires from the injected fluid, are probably the several causes contributing to the desired separation. Should the first injection not succeed, it may be repeated a second or third time. This injection is the more easy, from the structure of the umbilical vein,—having no valves, being extremely dilatable in all its branches, and forming altogether more than two-thirds of the substance of the placenta.

This suggestion of Dr. Mojon's having been communicated to many eminent practitioners, both in Italy and France, it has frequently been carried into effect, and, it is alleged, with great general success. *(Giornale Critico di Medicini Analitica.)*

MISCELLANEOUS.

*On Feigned Diseases of the Heart.* By Dr. QUARRIER.

The most formidable species of simulation which I have had to contend against has been the production of apparent organic disease of the heart, introduced into the royal marine artillery by George Chapman, who had been brought up under a veterinary surgeon, and had acquired a knowledge of the effects and noxious qualities of certain deleterious drugs. This practice was productive of some alarming consequences for a considerable period: some few were permanently injured, having actually produced the disease which they intended to counterfeit, and some others succeeded in obtaining their discharge, notwithstanding the vigilance of the medical officers at Haslar, where they were treated, and the strictest inspection on my own part.

While this man remained in the hospital, we were surprised to find an unusual number of cardiac affections, more particularly in the surgical wards where he was placed. Some who had gone into the hospital with slight gonorrhœa or syphilitic affections, had no sooner recovered from their original disease, than faintness, vomiting, and the utmost degree of nervous irritability, assailed them, leaving palpitations and a variety of dyspeptic feelings. Every curative means was adopted by the physicians and surgeons of the hospital, but with partial success: I say partial success, for some of them became tired of medical discipline, and got well speedily; but the rest continued until Chapman was sent out, when they got spontaneously well; a considerable degree of debility only remaining. I have already stated that Chapman deserted in a few days after being sent from Haslar. He was taken after an absence of some months, was punished, and sent again to Haslar hospital for cure; when, a fortnight had not elapsed before two cases of cardiac disease occurred in the ward where he was, and in men who had hitherto never exhibited the slightest symptom of such an affection. The symptoms assumed a considerable variety of form, with strong epigastric pulsation; and we could not doubt that means had been resorted to so as to produce this curious affection; but we were foiled in our various attempts at searches and surprises, and in discovering the particular means adopted to produce such anomalous symptoms. Chapman was again discharged cured, and very soon deserted a second time. He was taken in a remote part of the country, and succeeded in inducing the staff surgeon of the district militia, who had to examine him, to report his total incapacity for service, in consequence of *organic disease of the heart.* He had taken his medicine, and no one, unacquainted with the peculiar symptoms, and the expression of countenance, induced by the *helleborus albus*, could detect a person so practised in the art of simulating disease as Chapman was. He had found it profitable, and sold his powders to the seamen and marines at a considerable price, as well as receiving a gratuity upon success. The helleborus albus was the principal ingredient, and the powders were calculated either to produce a sudden and severe illness for the immediate occasion, or to gradually undermine the tone of the stomach and digestive organs, producing every appearance of dyspepsia, attended by great nervous irritability, and violent and continued palpitations! This drug, however, is most uncertain in its action, and it is constantly adulterated in the shops: the smaller doses, therefore, at times produced the most violent symptoms, and frequently deterred the person from persevering. It is fortunate that the adulteration was so great; for it will excite the astonishment of physicians, to know that two scruples, and sometimes a drachm, and even a drachm and a half, of this most noxious drug, was given as a full and effectual dose. The most violent symptoms were produced, threatening immediate dissolution; and I have reason to believe that two men, who came under my immediate inspection in 1821, must have died, had not remedies been immediately administered. Their cases made a serious impression; and, from the peculiar expression of countenance, we had acquired the readiest means of detection. (Mr. C. Hutchison's *Observations in Surgery*.)

